# A functional *in vitro* model of heterotypic interactions reveals a role for interferon-positive carcinoma associated fibroblasts in breast cancer

**DOI:** 10.1186/s12885-015-1117-0

**Published:** 2015-03-15

**Authors:** Abdel Nasser Hosein, Julie Livingstone, Marguerite Buchanan, James F Reid, Michael Hallett, Mark Basik

**Affiliations:** 1 Lady Davis Institute for Medical Research, Sir Mortimer B. Davis Jewish General Hospital, Montreal, Canada; 2 Department of Pharmacology & Therapeutics, McGill University, Montreal, Canada; 3 Department of Oncology, McGill University, Montreal, Canada; 4 Department of Surgery, McGill University, Montreal, Canada; 5 McGill Centre for Bioinformatics, Montreal, Canada; 6 Fondazione IFOM Istituto FIRC di Oncologia Molecolare, Milan, Italy; 7 Department of Oncology, Lady Davis Institute, 3755 Cote Ste Catherine, Montreal, QC H3T1E2 Canada

**Keywords:** Stroma, Carcinoma-associated fibroblasts, Breast cancer, Interferon

## Abstract

**Background:**

Cancer-associated fibroblasts (CAFs) play an important role in breast cancer pathogenesis by paracrine regulation of breast cancer cell biology. Several *in vitro* and mouse models have characterized the role of cell contact and cytokine molecules mediating this relationship, although few reports have used human CAFs from breast tumors.

**Methods:**

Primary breast CAF cultures were established and gene expression profiles analysed in order to guide subsequent co-culture models. We used a combination of colorimetric proliferation assays and gene expression profiling to determine the effect of CAFs on the MCF-7 breast cancer cell in an indirect co-culture system.

**Results:**

Using gene expression profiling, we found that a subgroup of breast CAFs are positive for a type one interferon response, confirming previous reports of an activated type one interferon response in whole tumor datasets. Interferon positive breast cancer patients show a poor prognostic outcome in an independent microarray dataset. In addition, CAFs positive for the type one interferon response promoted the growth of the MCF-7 breast cancer cell line in an indirect co-culture model. The addition of a neutralizing antibody against the ligand mediating the type one response in fibroblasts, interferon-β, reverted this co-culture phenotype. CAFs not expressing the interferon response genes also promoted the growth of the MCF-7 breast cancer cell line but this phenotype was independent of the type one fibroblast interferon ligand.

**Conclusions:**

Primary breast CAFs show inter-patient molecular heterogeneity as evidenced by interferon response gene elements activated in a subgroup of CAFs, which result in paracrine pro-proliferative effects in a breast cancer cell line co-culture model.

**Electronic supplementary material:**

The online version of this article (doi:10.1186/s12885-015-1117-0) contains supplementary material, which is available to authorized users.

## Background

Breast carcinoma is orchestrated by a complex series of molecular events and biological processes involving the contributions of several cell types [1,2]. Despite the fact that most of our understanding of cancer centers on those events taking place within the cancer epithelium, the cancer-associated stroma also plays a co-dominant role in shaping the biological and clinical fates of the disease [3,4]. Specifically, the carcinoma-associated fibroblast (CAF) has been shown to be a major player in the stroma’s influence on tumor growth [5,6]. Many reports have focused on the role that CAFs have in regulating TGF-β signalling and angiogenesis through secreted factors such as SDF-1 and VEGF [7-9]. CAFs have shown the ability to both promote [10] and repress [11] MCF-7 cell growth *in vitro*, in addition to having no effect at all [12]. Importantly, none of these studies took account of possible inter-patient CAF heterogeneity largely because, unlike tumor heterogeneity, little data exists about inter-patient CAF heterogeneity.

In particular, one *in vitro* model using a series of breast cancer cell lines directly co-cultured with normal human fibroblasts demonstrated that human fibroblasts will induce a type-one interferon response when admixed with tumorigenic breast cancer cell lines [13]. Furthermore, this type-one interferon response signature was shown to be expressed in a large proportion of breast tumors contained in the NKI breast tumor microarray dataset [14] and its expression in whole breast tumors was associated with a significantly poorer prognosis. In addition, this outcome was confirmed in an independent patient cohort in which immunohistochemical analysis of phospho-STAT1 was used as a proxy for the presence of the type-one interferon response.

In this report we show that there exists a subset of CAFs which express a type one interferon response which is stable upon *ex vivo* cultivation. This interferon response can impart a paracrine growth-promoting effect on the MCF-7 breast cancer cell line. Our findings suggest that an understanding of CAF molecular heterogeneity can be used to construct relevant preclinical *in vitro* models of tumor-stromal interactions.

## Methods

### Tissue culture

Primary tissue culture was carried out as previously outlined [15]. Briefly, invasive breast carcinoma specimens were surgically resected from patients at the Jewish General Hospital (Montreal, Canada). CAFs were determined to be intratumoral by a certified pathologist. Tissues were minced with a sterile blade and resuspended in a solution of DMEM with 10% fetal bovine serum (FBS) and 3% collagenase overnight at 37 degrees Celsius. The next day samples were filtered through an 8 μm mesh in order to remove undigested debris. The single cell suspension with viable fibroblasts was cultured in DMEM (10% FBS) for 2–3 weeks in a 24 well plate and then transferred to a T75 flask where it was continually maintained in a 2% FBS medium solution. All fibroblasts were harvested between passage doublings 3–5. Normal breast fibroblasts from reduction mammoplasties were collected at the same institution and in the same manner as the CAFs noted above. All fibroblast cultures underwent immunocytochemical analyses for pan-cytokeratin and vimentin as previously described by our group to confirm their mesenchymal identity [15]. All protocols involving human tissues were approved by the Research Ethics Committee of the Lady Davis Institute for Medical Research of McGill University and were in compliance with the Helsinki Declaration. Furthermore, all tissues procured from both reduction mammoplasty and tumor resection surgeries were obtained with the written informed consent of all patients.

### DNA microarray expression profiling

Gene expression profiling was carried out as described previously [15]. Briefly, fibroblasts were harvested from subconfluent cultures, cultivated in DMEM with 2% FBS. RNA was then extracted using the Mini RNA Extraction kit (Qiagen, Venlo, Netherlands). Five micrograms of total RNA was reversed transcribed with the Fairplay III Microarray Labeling kit according to the instructions of the manufacturer (Aglient Technologies, Santa Clara, California). The resulting cDNA was then precipitated with 70% ethanol, air-dried, resuspended in 5 μL of coupling buffer, and dissolved at 37°C for 15 minutes. Five microliters of Cy3 or Cy5 dye were added to the universal reference (Aglient Technologies) or fibroblast cDNA, respectively, and allowed to incorporate for 30 minutes at room temperature. Labeled cDNA was cleaned-up using Fairplay columns (Aglient Technologies) according to the instructions of the manufacturer. Labeled reference and fibroblast cDNA samples were combined and mixed with gene expression hybridization buffer and control targets supplied by the manufacturer and hybridized to a 4 × 44 K two-color whole human genome gene expression array for 17 hours at 65°C. The array was then washed in a solution of 6× SSPE, 0.005% *N*-lauroylsarcosine followed by a solution of 0.06× SSPE, 0.005% lauroylsarcosine and scanned on the Agilent DNA Microarray scanner at a resolution of 5 μm. All images were extracted and normalized with Feature Extraction software version 9.5. The microarray data from this study have been submitted to the NCBI Gene Expression Omnibus (GEO) (http://www.ncbi.nlm.nih.gov/geo) under accession number GSE29270.

### Analysis of fibroblast and Co-culture expression profiling data

Breast carcinoma derived fibroblasts were cultured and expression profiled as outlined previously by our group [15]. This dataset was analyzed using the straight-forward approach demonstrated by Sorlie and colleagues [16]: any gene that was two-fold above the median value for that gene in at least 3 patient samples was retained. Unsupervised clustering (Pearson’s correlation) was then performed using TIGR MeV version 4.1. In the case of the expression profiling on the MCF-7 breast cancer cell line, the three conditions (see below) were compared in a supervised 2 × 2 × 2 manner using the SAM algorithm [17]. The samples were then clustered in the same way as the unsupervised manner. This was also carried out by using TIGR MeV version 4.1.

### Interferon-β Enzyme Linked Immunosorbant Assay (ELISA)

Subconfluent fibroblast cultures were allowed to incubate in DMEM (2% FBS) for 48 hours at which time the medium was collected and spun down for five minutes at 1500 rpm in order to remove debris. An ELISA assay was carried out as per the manufacturer’s instructions using the Human Interferon-β kit (R&D Systems, Minneapolis, Minnesota).

### In vitro co-culture model

All experiments were carried out in DMEM media supplemented with 2% fetal bovine serum. 5000 MCF-7 cells (American Tissue Type Collection, Manassas, Virginia) were plated into flat bottom 24-well plates and allowed to adhere overnight. 600 μl of fresh media was then added and a semi-permeable insert with a 0.4 μm pore size (Millipore, Billerica, Massachusetts) was placed over the media. 5000 fibroblasts were then seeded into the insert, re-suspended in 400 μl of media for a total co-culture volume of 1 ml/well. Monoclonal antibodies (R&D Systems, Minneapolis, MN) were added at this time if necessary. After co-cultures had incubated for the appropriate time, inserts and fibroblasts were removed and MTT reagent (Sigma-Aldrich, St. Louis, Missouri) was added to the media (1:10 ratio) and allowed to incubate for 2 hours after which the media was aspirated and 1 ml of DMSO was added. The absorbance was measured at 570 nm. For RNA or protein harvesting, co-cultures were performed in 6-well dishes with appropriate transwell insert (Millipore) using 25,000 of each cell type in a total co-culture volume of 4 ml. After the appropriate co-incubation time, cells were snap frozen in liquid nitrogen for RNA harvesting.

### Quantitative Reverse Transcription Polymerase Chain Reaction (Q-RT-PCR)

Five micrograms of total RNA was reverse transcribed using Stratagene’s AffinityScript Multiple Temperature cDNA Synthesis Kit (Agilent Technologies) according to the manufacturer’s instructions. 1 μl of oligo(dT) primer was added to 5ug of total RNA and allowed to incubate at 65°C for five minutes. The reaction was subsequently cooled to room temperature. The following reactants were then added for a total volume of 20 μl : 2.0 μl of 10× AffinityScript RT Buffer, 0.8 μl of dNTP mix (25 mM of each dNTP), 0.5 μl of RNase Block Ribonuclease Inhibitor (40 U/μl) and 1 μl of reverse transcriptase. The reaction was carried out at 42°C for one hour and terminated by a 15 minute incubation at 70°C. The parameters for the interferon-associated Q-RT-PCR were adapted from Buess et al. [13]. PCR reactions were carried out in a final volume of 10 μl. Two micrograms of synthesized cDNA, 5 μl of 2× SYBR ®Green PCR Master Mix (ABI, Foster City, CA, USA) and 1 μl (10 μM ) of each primer (sequences: OAS2, forward GGAATACCTGAAGCCCTACGAA, reverse CCTGCAGACGTCACAGATGGT; IFNβ, forward ACCTCCGAAACTGAAGATCTCCTA, reverse TGCTGGTTGAAGAATGCTTGA; GAPDH, forward GAAGGTGAAGGTCGGAGTC, reverse GAAGATGGTGATGGGATTTC). Primers were purchased from Invitrogen (Carlsbad, California) and adapted from Buess et al. All reactions were carried out in an ABI 7700 Sequence Detection System using the following amplification conditions: 50°C for 2 minutes, 94°C for 10 minutes, followed by 40 cycles of 94°C for 15 s and 60°C for 60 seconds. All reactions were carried out in triplicate.

### NKI295 database analysis

Patients were split into high and low expressers of our IFN signature by hierarchical clustering. Hierarchical clustering was performed using Euclidean and Ward’s algorithm. A univariate Kaplan Meier analysis was then carried out in order to assess the prognostic significance of the IFN signature. Secondly, patients in the NKI patient cohort were split into two groups (high and low) based on their expression of S100A2 using hierarchical clustering. The low and high S100A2 expresser groups were separately clustered with the IFN signature. Kaplan Meier curves were subsequently generated.

## Results

### Gene expression profiling reveals the presence of a CAF subtype that is positive for a type one interferon response

**Figure 1:**
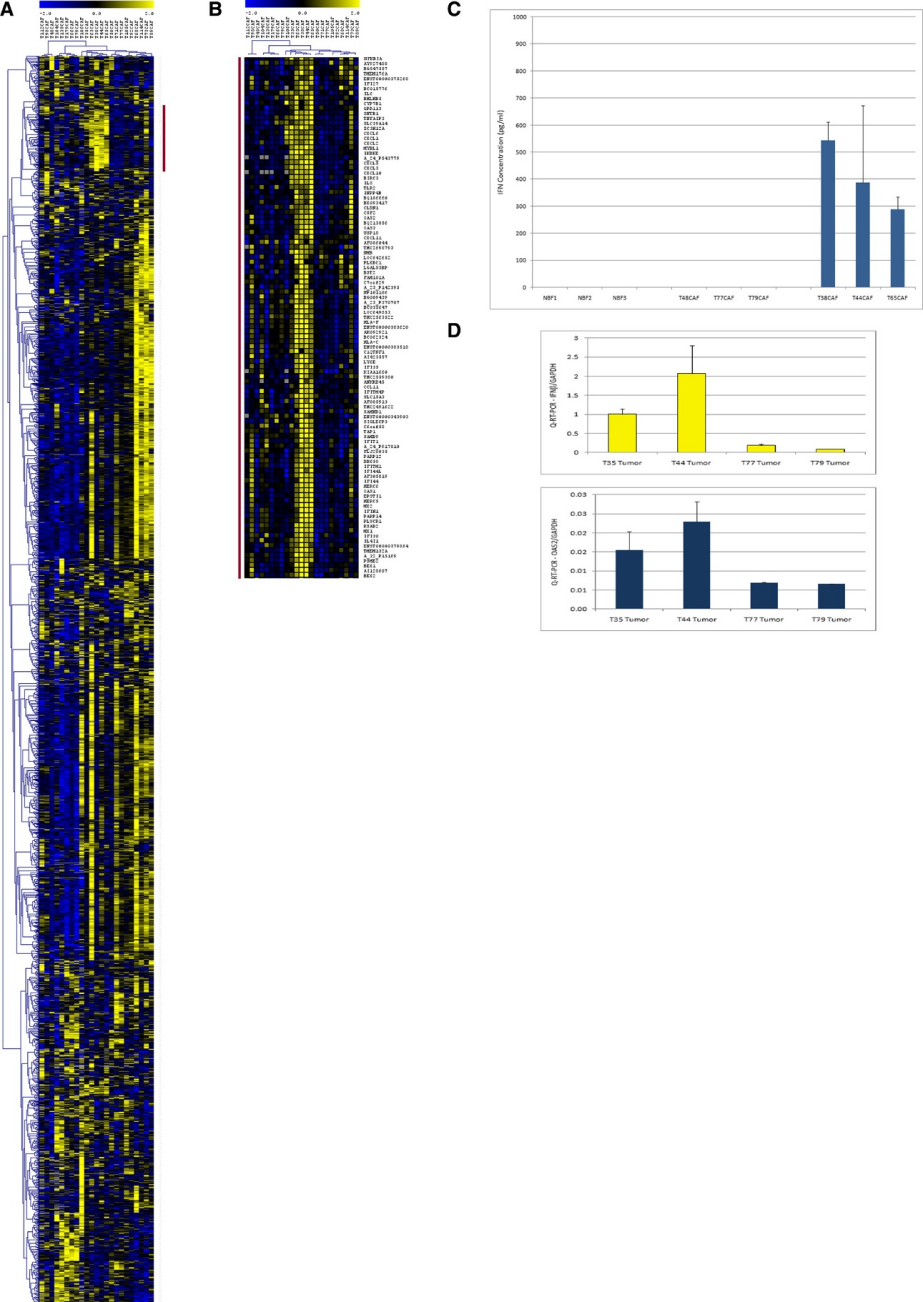
**Hierarchical clustering of CAFs reveals interferon positive CAF subset. (A)** Hierarchical clustering (HCL) of the 23 CAFs. DNA microarray data were filtered for genes that were 2-fold above or below the mean for that gene in a minimum of 3 samples. This resulted in a total of 2506 genes which are shown clustered above. **(B)** Magnification of the gene cluster highlighted by the red bar in Figure 1A. Further inspection of this cluster shows the upregulation of many type-one interferon genes in addition to a host of cytokines in five of the CAFs analyzed. **(C)** An IFN-β ELISA of DMEM (2% FBS) cultured with sub-confluent fibroblasts (3 normal breast fibroblast, 3 IFN-negative CAFs and 3 IFN-positive CAFs) for 48 hours. IFN-β ligand was only detected in the 3 IFN-positive CAF supernatents. **(D)** Q-RT-PCR analysis on frozen whole tumor sections corresponding to two IFN-positive and two IFN-negative CAFs. The IFN-positive CAFs (T35 and T44) showed a greatly increased level of both IFN markers IFN-β and OAS2 relative to the IFN-negative tumors (T77 and T79) (p < 0.001, by way of Bonferroni Multiple comparison test following a one-way ANOVA).

**Figure 2:**
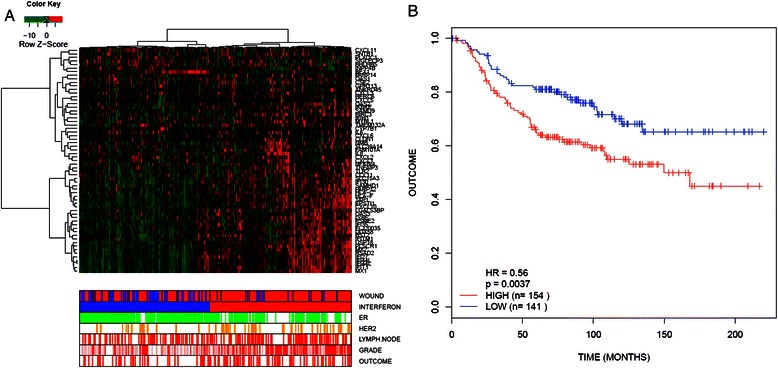
**Interrogation of the NKI295 breast cancer microarray dataset for the clinical significance of the IFN signature shown in Figure**1**. (A)** the IFN signature was able to divide the cohort into two groups; one consisting of 154 patients (high IFN expressers) and the second consisting of 141 patients (low IFN expressers). Bars at the bottom of the figure indicate various histopathological characteristics of the tumors/patients whose microarray data appear in the corresponding column. ‘Outcome’: red denotes recurrence, white denotes no recurrence. ‘Grade’ refers to the histological grade of the breast carcinoma: red is grade 3, pink is grade 2 and white is grade 1. ‘Lymph node’: red indicates that the patient has axillary lymph node dissemination of the breast carcinoma, whereas white is negative for lymph node dissemination. ‘HER2’: orange indicates that the patient’s carcinoma was positive for HER2 over-expression, and white indicates negativity. ‘ER’: green indicates estrogen positive disease and white indicates estrogen receptor negative disease. ‘Interferon’: red are patients who cluster in the interferon positive group and blue are patients who do not overexpress the interferon cluster of genes. ‘Wound’: red denotes patients who are positive for the wound response signature of Chang et al. [19] and blue are those that do not overexpress the wound response. **(B)** These two distinct groups had a significantly different outcome with the high expressers of IFN displaying a greater rate of recurrence than the low IFN expressers.

We carried out gene expression profiling of primary breast CAFs. This dataset consisted of 23 patient-derived CAFs that were cultured for a minimum of 3 doublings and a maximum of 6 doublings in low serum conditions. In these analyses the data were filtered for the most variably expressed genes [16,18]; any gene that was 2-fold above or below the mean for that gene in at least 3 of the 23 samples was retained. This strategy yielded a filtered list of 2506 genes. Upon hierarchical clustering the CAF cohort was clearly subdivided into two distinct groups (Figure 1A). Upon closer inspection it was evident that a group of 5 CAFs (T35, T63, T38, T44, T65) clustered tightly together and that this pattern was largely due to the overexpression of a coordinated gene cluster (Figure 1B). This expression block consisted of 101 genes and has members such as MX1, MX2, OAS1, OAS2, IFI27 and IFI30 greatly overexpressed within it, compared to the other CAFs, strongly suggesting that it represents an activated type one interferon response pattern. The type one interferon mediators were present in addition to several cytokine and chemokine transcripts (Figure 1B). Quantitative RT-PCR analysis of two key interferon response genes, MX1 and OAS2, validated microarray gene expression results (Additional file 1: Figure S1) showing significantly higher expression levels in CAFs found within the activated interferon response cluster. In addition, Q-RT-PCR analysis of the IFN-β gene was correlated with RNA expression of MX1, suggesting that this particular type one interferon response was likely due to interferon-β (IFN-β) ligand expression (Additional file 2: Figure S2). Specifically, by Q-PCR, the five highest expressers of IFN-b were T65, T63, T73, T44, T38 which had an average MX1/GAPDH QPCR ratio of 3.19 versus a ratio of 0.05 in the rest of the cohort (p = 0.005). Four of these five CAFs grouped closely in the microarray clustering data with the one that did not (T73CAF) still showing positivity for much of the IFN gene expression cluster (Figure 1). Next, by way of ELISA, we confirmed the presence of the IFN-β ligand in the tissue culture supernatants of activated IFN-response CAFs and lack of the ligand in normal breast fibroblasts and IFN-negative CAFs (Figure 1C). We then found that RNA extracted from two whole tumor sections whose CAFs were deemed negative for the type one interferon response via microarray and Q-RT-PCR analyses, showed considerably lower levels of both IFN-β and OAS2 as compared to whole tumors from two patients from whom activated interferon response CAFs were obtained (Figure 1D). These findings suggest that the interferon response we observed in our CAFs was unlikely due to an *ex vivo* tissue culture artefact. Finally, we used our 101 gene CAF-derived cytokine-enriched interferon signature to interrogate the NKI295 breast cancer microarray dataset (Figure 2). Consistent with Buess et al’s findings, our cytokine enriched interferon signature was deemed to be over expressed in 154 of the 295 patients and patients with tumors showing such over-expession showed a worse outcome than those with tumors that fell into the interferon negative group (hazard ratio = 0.56, p = 0.0037).

### Carcinoma-associated fibroblasts impart pro-cancer effects on the MCF-7 breast cancer cell line in an indirect heterotypic co-culture system

**Figure 3:**
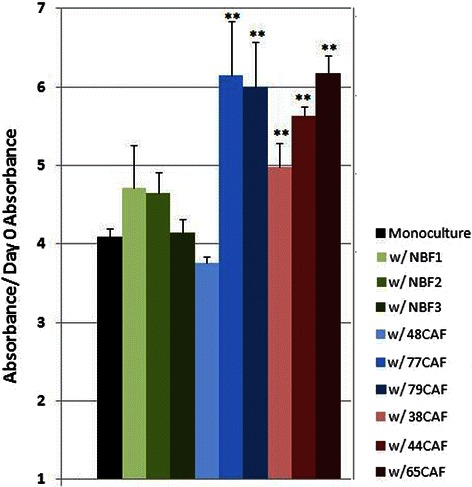
**MCF-7-CAF co-culture phenotype.** Three types of fibroblasts were involved in co-cultures with the MCF-7 breast cancer cell line. Normal breast fibrobasts are denoted in green, IFN-negative CAFs in blue and IFN-positive CAFs in red. All experiments were performed in triplicate. A single 120 hour time point is shown. Statistical differences were ascertained by an analysis of variance. Double asterisks represent samples that were deemed to be significantly different (p < 0.01) from the MCF-7 grown in mono-culture (post-hoc test: Dunnett’s multiple comparison test). Five of six CAFs showed significant growth promotion of the MCF-7 breast cancer cell line in this model.

In order to test the effects of IFN-positive CAFs on the growth of breast cancer cells we developed an *in vitro* co-culture model in which fibroblasts were indirectly co-cultured with the MCF-7 breast cancer cell line for 120 hours. The fibroblasts and MCF-7 cells were separated by a semi-permeable membrane (pore size of 0.4 μm) with the MCF-7 cells being cultured on the bottom layer. At the desired time point, the transwell insert containing the fibroblasts was discarded and the MCF-7 s were either assayed by way of the MTT cell viability reagent or harvested for RNA and/or protein. Fibroblasts from three of each type of patient (IFN-negative, IFN-positive and normal reduction mammoplasty) were co-cultured with the breast cancer cell line. We found that co-culturing CAFs with MCF-7 breast cancer cells increased the proliferation rate of the latter, unlike co-culturing with normal breast fibroblasts (NBF) (Figure 3). Interestingly, one of the IFN-negative CAFs (T48CAF) was not capable of promoting MCF-7 proliferation, while the other two IFN-negative CAFs increased MCF-7 cell proliferation to an equal or greater degree as compared to the three IFN-positive CAFs.

### The pro-MCF-7 effects by IFN-positive CAFs are dependent on the continued action of the IFN-β ligand whereas this is not a requirement for the pro-MCF-7 effects of the IFN-negative CAFs

**Figure 4:**
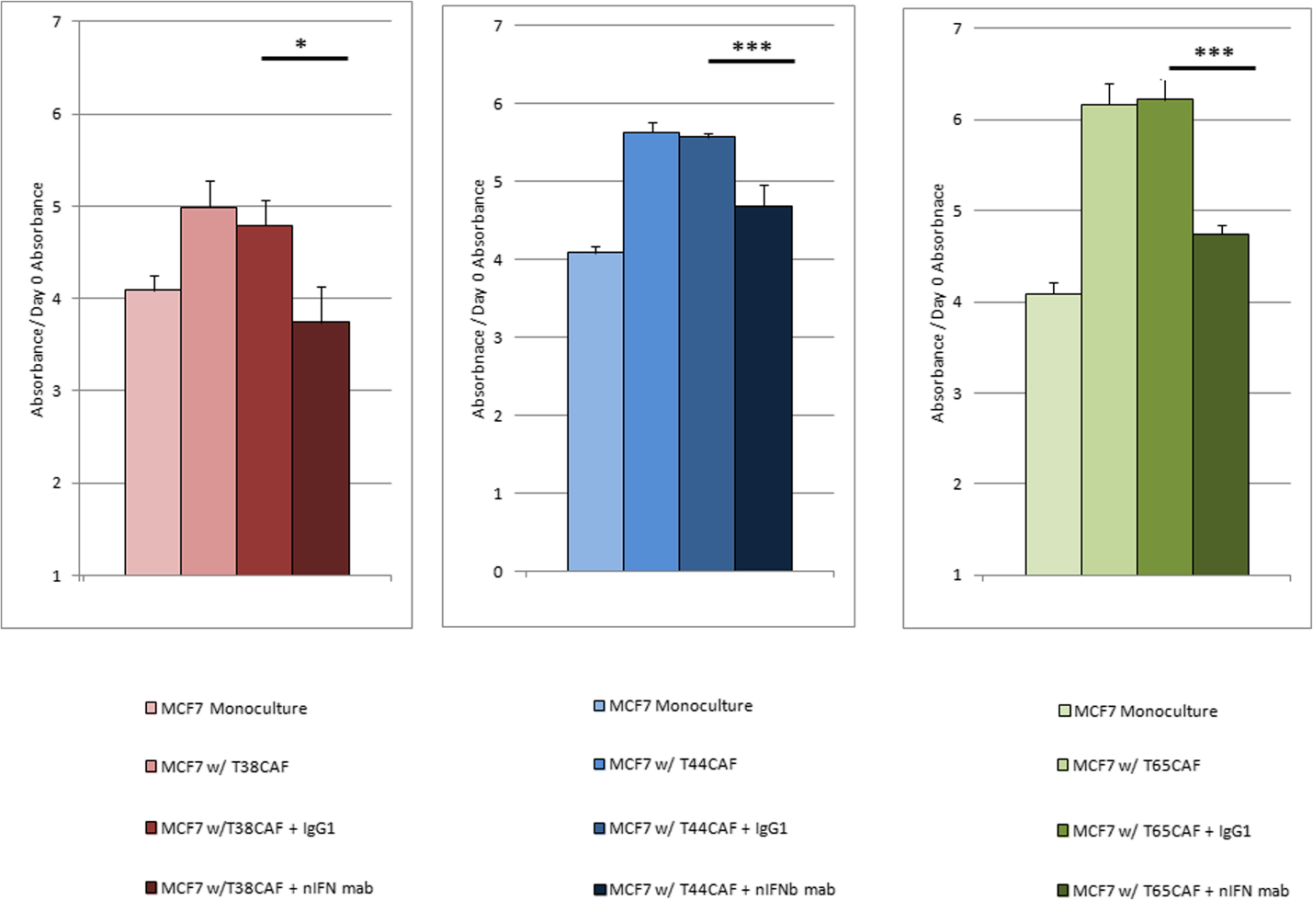
**IFN-β is responsible for the positive effect of IFN-positive CAFs on MCF-7 proliferation.** Three IFN-positive CAFs, T38, T44, T65, were co-cultured as previously described. In this experiment, two new conditions where added: once triplicate for each day was reserved for the addition of 20 μg of an IgG_1_ isotype control antibody (second darkest shade) and another for 20 μg of neutralizing IFN-β antibody (darkest shade). A Bonferonni multiple comparison test was carried out after a one-way ANOVA was performed on the four conditions at 120 hours. The asterisks represent the significant difference between the co-culture with the IgG isotype control and the co-culture with the neutralizing IFN-β antibody (*, p < 0.05; ***, p < 0.001).

To determine if the type-one interferon response in CAFs is responsible for their ability to promote MCF-7 growth, we repeated the co-culture proliferation assays in the presence of a neutralizing monoclonal antibody against the IFN-β ligand in the co-culture medium (Figure 4). When IFN-β antibody was added to the culture medium, all 3 co-cultures involving the IFN-positive CAFs demonstrated a significant reversion of the phenotype, to proliferation levels very near that of MCF-7 cells cultured in the absence of CAFs. Thus the pro-proliferative effect on MCF-7 cells of the IFN-positive CAFs is dependent on the presence of IFN-β. Of note, the addition of the anti-IFN-β antibody did not result in reversal of growth promoting effects in co-cultures with IFN-negative CAFs (Additional file 3: Figure S3).

### Microarray analysis of the IFN-positive-CAF-MCF-7 co-cultures reveals the presence of the candidate tumor suppressor gene S100A2 as a putative mediator of the pro-cancer heterotypic phenotype

**Figure 5:**
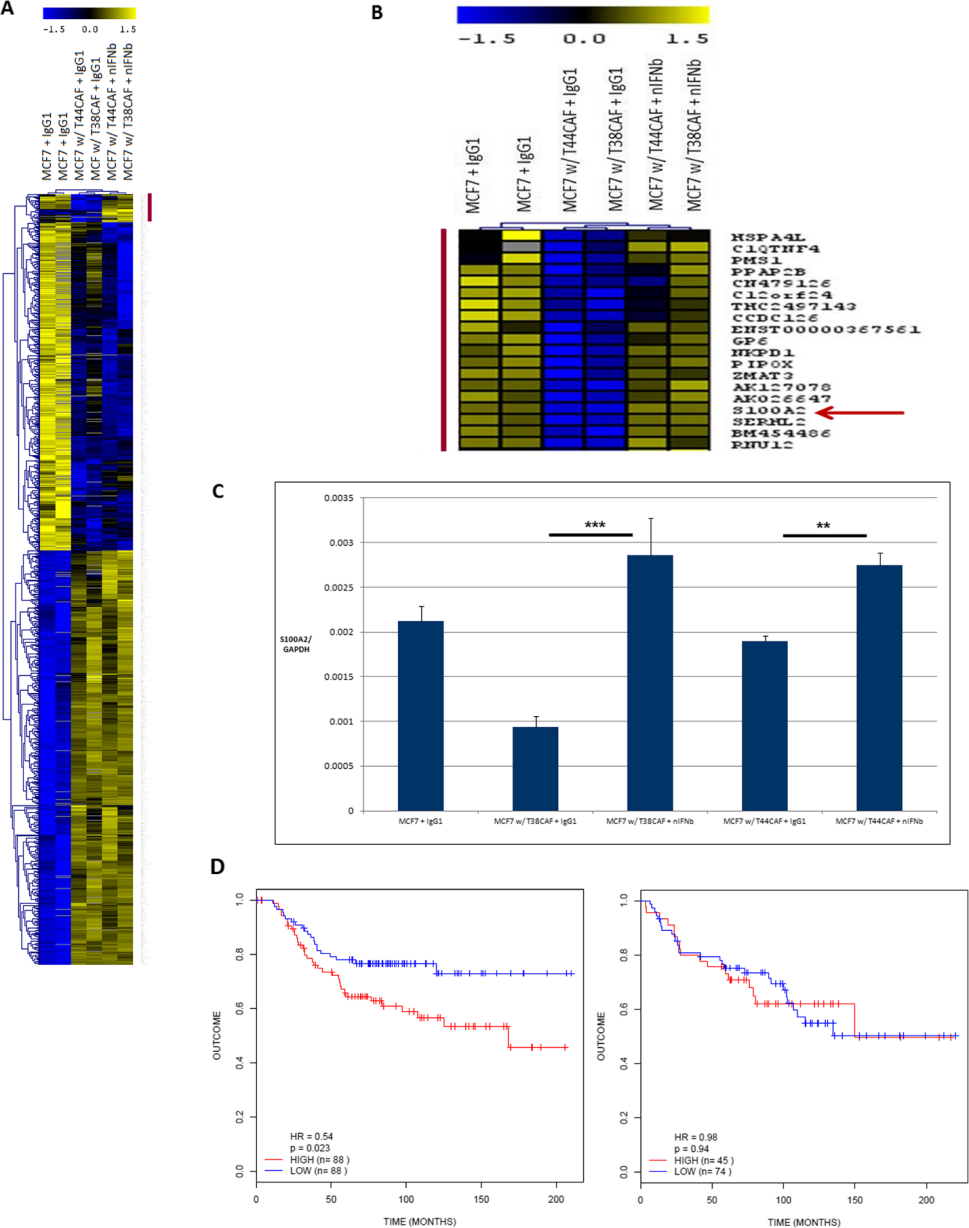
**Gene expression analysis shows that S100A2 expression in MCF-7 cells is modulated by the activity of IFN-positive CAFs. (A)** A 2 × 2 × 2 SAM analysis was carried out to compare MCF-7 s under three different conditions: MCF-7 monocultured with only the IgG_1_ antibody, MCF-7 co-cultured with two IFN-positive CAFs and the IgG_1_ antibody, and MCF-7 co-cultured with the same two IFN-positive CAFs in addition to the neutralizing IFN-β monoclonal antibody. 995 modulated genes and corresponding clustering are displayed. **(B)** The 19 transcripts that are significantly different between the second condition and both the first and third conditions are magnified. S100A2 (red arrow) is highlighted. **(C)***S100A2*’s differential mRNA expression was confirmed by Q-RT-PCR. An ANOVA analysis followed by a Bonferonni multiple comparison test was performed to show that *S100A2* expression rose when the IFN-β neutralizing antibody was added to the IFN-positive co-culture. **(D)** The Kaplan Meir analysis on the NKI295 cohort when *S100A2* expression is low (left) shows that IFN-positive patients (red) have a significantly shorter survival duration when compared to IFN-negative (blue) patients. Conversely, when *S100A2* expression is high (plot on right), the IFN-positive and negative groups have statistically indistinguishable survival profiles.

In order to gain a greater understanding of the molecular mediator(s) of the pro-proliferative effects of the IFN-positive CAFs, we carried out gene expression profiling of the MCF-7 breast cancer cell line under various co-culture and mono-culture conditions. The MCF-7 breast cancer cell line was grown alone or co-cultured with the IFN-positive CAFs T38 and T44, in the presence of IgG_1_ or neutralizing IFN-β monoclonal antibodies for 72 hours at which point RNA was harvested from the breast cancer cell line. We carried out a supervised clustering approach comparing three different groups of samples in a 2x2x2 study design; MCF-7 breast cancer cell line with IgG_1_ isotype control antibody, MCF-7 co-cultured with the two IFN-positive CAFs and the IgG_1_ antibody and lastly, MCF-7 co-cultured with the two IFN-positive CAFs and treated with the IFN-β neutralizing antibody. There were a total of 995 differentially expressed genes when a false discovery rate of 5% was applied to the SAM analysis (Figure 5A). Notably, the overwhelming majority of differentially expressed genes were between mono-culture and co-culture conditions. There was however a group of genes that was significantly down-regulated under co-culture conditions but reverted back to higher expression levels when the IFN-β neutralizing antibody was added to the co-culture. This group consisted of 19 transcripts of which 12 were annotated genes. Given that the lower expression of these genes correlated with the pro-growth phenotype (Figures 4 and 5A) we reasoned that any putative effector gene in this cluster should function in a tumor-suppressor-like manner if it is to modulate the phenotype we observed. One of these genes , *S100A2*, had already been identified as a putative tumor suppressor in breast as well as in other types of cancers [20,21]. These microarray finds were confirmed by way of Q-RT-PCR (Figure 5C). We then interrogated the NKI295 database in order to evaluate the S100A2 gene as a univariate predictor; there was no correlation between outcome and expression levels (data not shown). Next, we separated the entire cohort into the respective high and low expressers of *S100A2* according to the median values of *S100A2* expression, and recalculated the Kaplan-Meir curves based on our interferon signature. In the low *S100A2* group (Figure 5D, left), the high and low IFN expressers were split precisely in half (n = 88 in each group), with the high expressers of the IFN signature showing a poorer prognostic outcome as compared to the low expressers (HR = 0.54, p = 0.023), consistent with our previous analyses. However, when S100A2 was relatively overexpressed this poor outcome associated with the interferon response was not observed, with the high and low expressers of the interferon signature showing a statistically indistinguishable survival outcome (Figure 5D, right). In addition, regarding the molecular subtypes of breast cancers in the NKI295 database, the basal and HER2 positive subtypes carry a significantly increased proportion of IFN positive patients versus the other three classical subtypes (chi squared = 82.8539, df = 4, p-value < 2.2e-16). Interestingly, even after segregating the cohort based on IFN status, which enriches for HER2 and basal poor outcome patients, S100A2 status is still able to demonstrate survival differences in the cohort (Figure 5D). Additional file 4: Figure S4 shows the overlap in molecular subtype, IFN status and S100A2 status in the NKI295 cohort. These above analyses are consistent with our *in vitro* findings (Figure 5A and B) suggesting that lower S100A2 expression is associated with the pro-tumorigenic effects of the type one interferon response within the breast cancer microenvironment.

## Discussion

The tumor microenvironment has been recognized as a major player in the development and progression of solid tumors, including breast cancer. Recently, targeting immune cells within the tumor microenvironment has led to spectacular successes in the treatment of melanomas [22,23]. The role of interferons in modulating the immune response to viruses is well known, but the role of interferons in modulating the immune response to tumors is less well defined. Early experimental models have uncovered potent direct cytotoxic and/or anti-proliferative effects of interferons [24-27], although the translation of these findings into their use as anti-cancer therapeutics [28-30] has been met with only limited success in breast cancer [30] and other carcinomas [31]. In fact, there were even early indications that the administration of IFN-β to cancer patients could lead to an increase in the number of hormone receptors in the cancerous tissue [32]. More recently, the presence of aberrantly expressed IFN-related genes in cancer were first noticed in the initial molecular portraits of breast cancer [18]. Later an IFN signature was observed in several human cancers; 15% of childhood lymphoblastic leukemias, 20% of ovarian and 40% of breast cancers were positive for an IFN-related signature [33].

In the current study we have shown that a subset of breast CAFs (5 of 23 tested CAFs) strongly expresses a type one interferon response and that this response, chiefly through the IFN-β cytokine, can impart a pro-proliferative effect on MCF-7 breast cancer cells *in vitro.* It should be noted that direct fibroblast-breast cancer cell line contact was necessary when the interferon response was previously induced artificially *in vitro* [13]. We show that an interferon response is identifiable even after *ex-vivo* culturing in some CAFs grown alone, and that its pro-proliferative effect on co-cultured breast cancer cells is mediated through the action of soluble IFN-β ligand. Our IFN response can be detected in whole breast tumors as an expression signature conveying poor prognosis. Additionally, we showed that S100A2 is a candidate mediator of the IFN response’s effect on patient outcome. S100A2 is a calcium binding protein that has been repeatedly shown to be down regulated in a variety of cancers such as breast [34], and prostate [35] and is considered to be a candidate tumor suppressor gene.

In light of recent findings that interferon positivity correlates with a poor clinical outcome in breast cancer [13], it is probable that interferons may actually be pro-tumorigenic, as suggested also by our findings. Type one interferon signaling has also been correlated with resistance to doxorubicin and topoisomerase-II inhibitors *in vitro* [36] and confer resistance to DNA damage in cancer cell lines [37]. Taken together, this would suggest that type one interferon neutralization within the tumor microenvironment should be pursued in lieu of their supplementation at least in cases in which interferon signalling is active and detectable in the tumor microenvironment [6]. Moreover, on close inspection of the interferon response gene set we have identified there are many cytokines present (CXCL1, CXCL2, CXCL6, CXCL10, CXCL11, IL6, IL8, CSF2, CCL11), any one of which could be mediating the phenotype seen herein.

## Conclusion

We have identified a subset of CAFs and perhaps breast tumors, which may be particularly vulnerable to such therapeutic approaches. These data will need to be further expanded to include *in vivo* models of human CAFs co-implanted with breast cancer cell lines. Taken together, these results provide a better understanding of the potential value of targeted anti-IFN-β therapy in breast cancer patients whose tumors show a gene expression profile reflecting a type-one IFN response. Protein biomarkers such as S100A2, OAS2 and/or IFNβ RNA expression in breast tumors may prove to be useful guides in predicting the response of IFN-positive patients to anti-interferon therapeutics.

## Additional files

Additional file 1: Figure S1. Q-RT-PCR corroboration of the type-one interferon signature revealed by way of microarray analysis. Microarray values for the genes MX1 (left) and OAS2 (right) are shown to significantly correlate with Q-RT-PCR values with R^2^ values of 0.825 and 0.445 respectively.
Additional file 2: Figure S2. Q-RT-PCR analysis of fibroblast interferon (IFN-β) versus the MX1 Q-RT-PCR values indicating a significant role of IFN-β in this interferon response.
Additional file 3: Figure S3. All three IFN-negative CAFs’ were co-cultured in the presence of the IFN-β neutralizing antibody and a decrease in MCF-7 proliferation was not observed. A single time point (120 hours) is shown and all co-culture conditions are compared to the MCF-7 mono-culture absorbance readings.
Additional file 4: Figure S4. Overlap between S100A2, IFN status and molecular subtype of breast cancer in the NKI295 database. Black denotes positivity for S100A2 and IFN status. Regarding molecular subtype: dark blue: luminal A, light blue: luminal B, red: basal, pink: her2, green: normal-like. See results section for further commentary.
