# Supplementary figures and images for: A functional in vitro model of heterotypic interactions reveals a role for interferon-positive carcinoma associated fibroblasts in breast cancer
Source: BMC Cancer. 2015 Mar 15;15:130. doi: 10.1186/s12885-015-1117-0 (PMC4369836; doi:10.1186/s12885-015-1117-0)

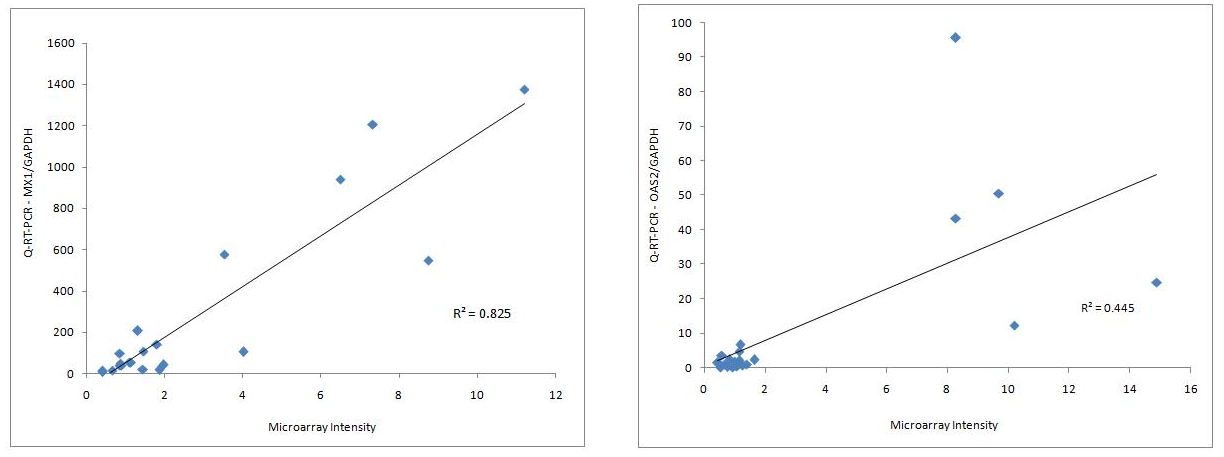

Supplement: Additional file 1: Figure S1. — Q-RT-PCR corroboration of the type-one interferon signature revealed by way of microarray analysis. Microarray values for the genes MX1 (left) and OAS2 (right) are shown to significantly correlate with Q-RT-PCR values with R2 values of 0.825 and 0.445 respectively. [file 12885_2015_1117_MOESM1_ESM.tiff]

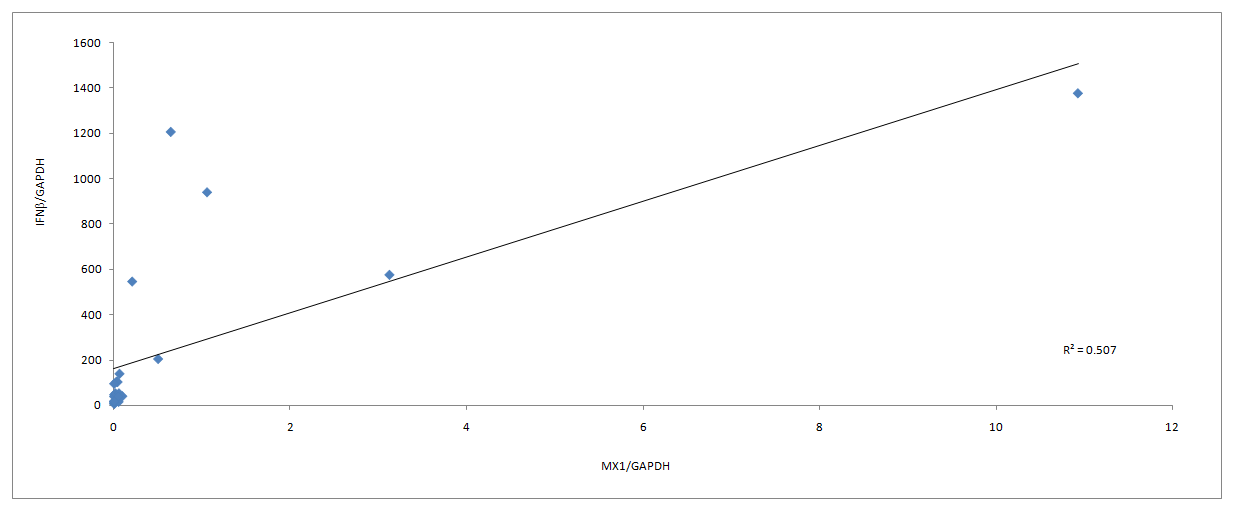

Supplement: Additional file 2: Figure S2. — Q-RT-PCR analysis of fibroblast interferon (IFN-β) versus the MX1 Q-RT-PCR values indicating a significant role of IFN-β in this interferon response. [file 12885_2015_1117_MOESM2_ESM.tiff]

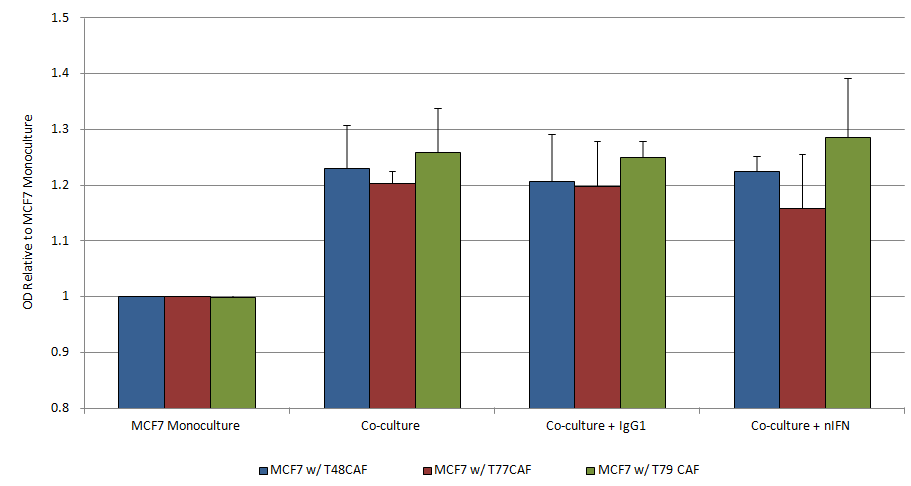

Supplement: Additional file 3: Figure S3. — All three IFN-negative CAFs’ were co-cultured in the presence of the IFN-β neutralizing antibody and a decrease in MCF-7 proliferation was not observed. A single time point (120 hours) is shown and all co-culture conditions are compared to the MCF-7 mono-culture absorbance readings. [file 12885_2015_1117_MOESM3_ESM.tiff]

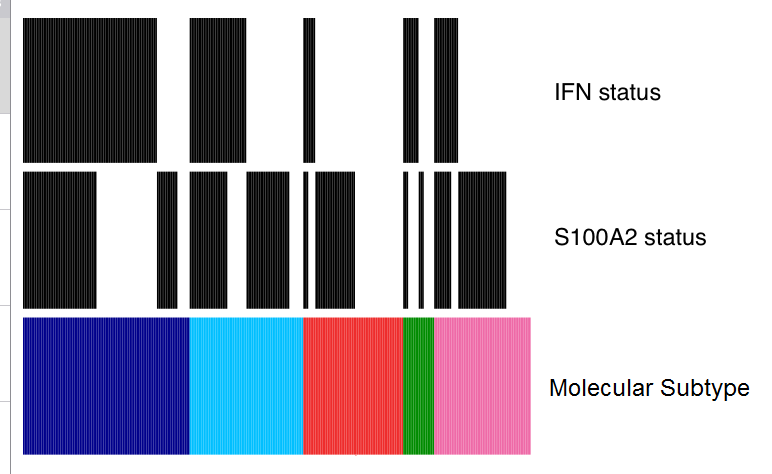

Supplement: Additional file 4: Figure S4. — Overlap between S100A2, IFN status and molecular subtype of breast cancer in the NKI295 database. Black denotes positivity for S100A2 and IFN status. Regarding molecular subtype: dark blue: luminal A, light blue: luminal B, red: basal, pink: her2, green: normal-like. See results section for further commentary. [file 12885_2015_1117_MOESM4_ESM.tiff]
